# Role of Valganciclovir in Children with Congenital CMV Infection: A Review of the Literature

**DOI:** 10.3390/children10071246

**Published:** 2023-07-19

**Authors:** Davide Pata, Danilo Buonsenso, Arianna Turriziani-Colonna, Gilda Salerno, Lucia Scarlato, Lara Colussi, Rolando Ulloa-Gutierrez, Piero Valentini

**Affiliations:** 1 Global Health Research Institute, Istituto di Igiene, Università Cattolica del Sacro Cuore, 00168 Rome, Italy; davide.pata01@gmail.com; 2 Department of Woman and Child Health and Public Health, Fondazione Policlinico Universitario A. Gemelli, 00168 Rome, Italylucia.scarlato.93@gmail.com (L.S.); piero.valentini@unicatt.it (P.V.); 3 Medicine and Surgery, Università Cattolica del Sacro Cuore, 20123 Rome, Italy; 4 Servicio de Infectologia Pediatrica, Hospital Nacional de Niños “Dr. Carlos Sáenz Herrera”, Centro de Ciencias Médicas, Caja Costarricense de Seguro Social (CCSS), San José P.O. Box 1654-1000, Costa Rica; rolandoug@gmail.com; 5 Instituto de Investigación en Ciencias Médicas UCIMED (IICIMED), San José 10108, Costa Rica; 6 Cátedra de Pediatría, Facultad de Medicina, Universidad de Ciencias Médicas (UCIMED), San José 10108, Costa Rica

**Keywords:** cCMV, Valganciclovir, children, SNHL, neutropenia

## Abstract

Background: Cytomegalovirus (CMV) is the most common agent of congenital infection in humans. It is a main cause of neurodevelopmental delay and sensorineural hearing loss in infancy. Since the 2000s, a number of studies have used Valganciclovir as a therapy for children with congenital CMV infection. Methods: In order to evaluate the efficacy of Valganciclovir in preventing clinical sequelae and its possible side effects, we performed a review of the published literature. This search was completed via PubMed for manuscripts published from January 2007 to December 2021, combining the MeSH words “Valganciclovir”, “Congenital”, and “Cytomegalovirus”. Results: A total of 27 articles were included (12 retrospective studies, 4 prospective studies, 1 randomized controlled trial, and 10 case reports). The clinical features were similar to those already described in the literature. The therapeutic protocols used were very different between the various studies included and neonatal antiviral treatments were only moderately effective. The therapy proved to be well-tolerated. Conclusions: The quality of the included studies and the sample size were limited due to the rarity of the disease. The use of different therapeutic protocols in terms of starting dates, doses, and durations made it impossible to compare and correctly evaluate the efficacy of the treatments. Randomized controlled trials are needed to establish the correct effective dose with the fewest side effects and the most efficient duration of therapy.

## 1. Introduction

Cytomegalovirus (CMV) is the most common agent of congenital infection in humans, accounting for an overall birth prevalence of 0.64% [1]. It is a main cause of neurodevelopmental delay and non-genetic sensorineural hearing loss (SNHL) in infancy [2].

When CMV is transmitted during pregnancy, the congenital infection (cCMV) is symptomatic in approximately 10–15% cases, and 90% of the newborns are asymptomatic but might develop late sequelae, especially sensorineural hearing loss (SNHL) [3,4,5]. Hence, the visual, neurological, and audiological follow-up of newborns infected in utero is of great importance.

Efforts have been made to establish a treatment regimen and to define when and whether a baby needs to be treated. Multiple studies exist on providing symptomatic babies with antiviral therapies, while there is a lack of evidence for asymptomatic newborns.

We performed a review of the literature published from 2007 to 2021 to collect the evidence about antiviral therapies for congenital CMV infections. Currently, two different antivirals are in use: oral valganciclovir (VGC) and intravenous ganciclovir (GCV). VGC is actually the drug of choice because of its oral administration.

The aim of our study was to analyze the available data on the VGC treatment of symptomatic and asymptomatic cCMV babies and its safety and efficacy in reducing symptoms and sequelae. 

## 2. Materials and Methods

We performed a narrative literature review to evaluate the efficacy of Valganciclovir in infants with cCMV infection to prevent clinical sequelae and its possible side effects. We considered the suggestions of the PRISMA group [6]. 

### 2.1. Search Strategy

Our literature search strategy was aimed at evaluating the benefits and side effects of VGC treatment in cCMV infection.

The PICOS approach was used to carry out our research as follows: population and infant patients with congenital CMV infection; intervention and comparison and study of the clinical features of children included in the study; outcomes and identification of the possible benefits and side effects of the therapy; and study design (all studies of quantitative research while protocols, letters to the editor, and reviews were excluded).

A search of PubMed was performed for the period January 2007 to December 2021, combining the MeSH words “Valganciclovir”, “Congenital”, and “Cytomegalovirus”.

### 2.2. Eligibility Criteria and Identification of Studies

We included in our review only studies aimed at evaluating the use of VGC in cCMV infection.

Clinical trial, case reports, and observational cohort studies—prospective or retrospective—were selected, while we excluded manuscripts without full free text, those which were not in English, studies with different goals that did not comprise quantitative research, and studies including only the use of GCV.

### 2.3. Study Selection

All studies published between January 2007 and December 2021 were considered (*n* = 174). Three senior reviewers, in order to increase consistency, reviewed the same publications and modified the screening and data extraction. The same reviewers evaluated the abstracts and full text of all identified publications using an online platform (covidence.org) to include studies and extract data. Any disagreements were resolved with the advice of other reviewers, if necessary.

### 2.4. Data Extraction

Two reviewers independently analyzed data from each included study relating to clinical characteristics, reported outcomes, and side effects. A third researcher verified the results across the original manuscripts.

### 2.5. Data Synthesis

Data from the included (and excluded) studies were entered in tabular form on an excel spreadsheet. They were presented in columns as follows: study citation, year, study country, type of study, number of patients involved, years of follow-up, clinical features, side effects, and outcomes. We aimed to describe the VGC protocols used, side effects, and outcomes for children treated with VGC.

## 3. Results

**Figure 1:**
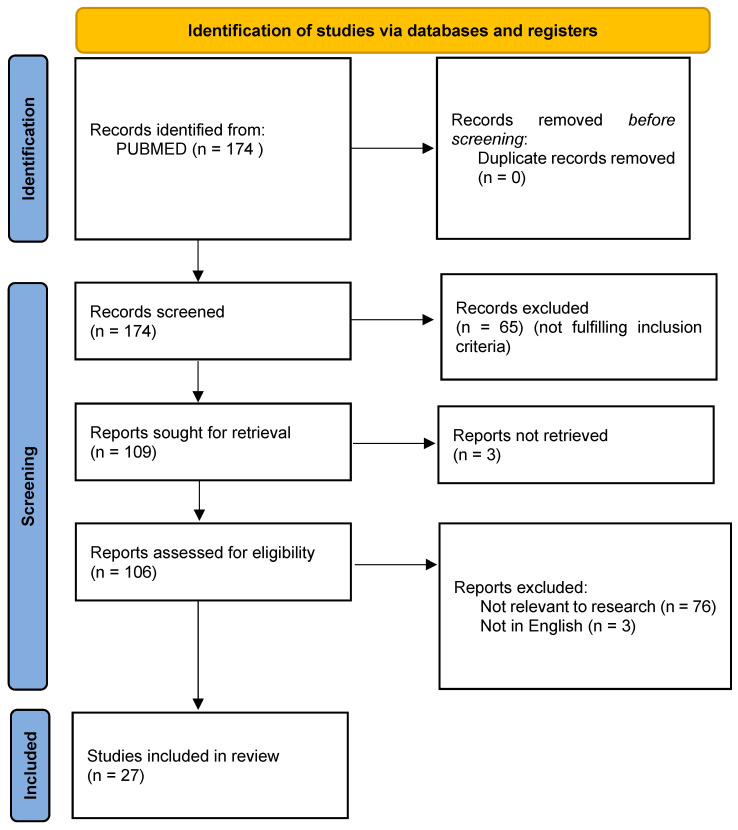
PRISMA 2020 flow diagram for the included studies.

We initially imported 174 studies (Figure 1), and 65 were excluded after reading the titles and abstracts. After evaluation of the full texts, an additional 82 manuscripts were deleted as 76 were not relevant to our study, 3 were not in English, and 3 were not available.

A total of 27 articles were included [7,8,9,10,11,12,13,14,15,16,17,18,19,20,21,22,23,24,25,26,27,28,29,30,31,32,33], comprising 12 retrospective studies, 4 prospective studies, 1 randomized controlled trial, and 10 case reports (Table 1).

### 3.1. Clinical Features (Table 2)

All included studies reported the clinical characteristics of children with cCMV, except for the study by Stronati et al. [29].

The most frequently described characteristics were brain abnormalities [7,8,9,10,11,13,14,15,16,18,19,20,21,22,25,26,28,30,31,32,33] and SNHL [7,11,12,13,14,15,16,17,19,20,21,22,24,25,26,27,28,30,31,32,33]. A small number of studies detected intra-uterine growth restriction (IUGR) [7,11,13,14,17,18,20,21,22,23,25,31,32,33], microcephaly [7,11,13,14,17,20,21,22,25,26,31,33], thrombocytopenia [7,8,9,11,14,20,21,22,23,24,25,26,28,32,33], and hepatitis [7,9,11,12,14,15,20,21,22,23,24,25,26,28,32,33].

Retinitis [7,14,20,21,25,26,33], prematurity [7,8,11,13,17,20,23,25,27,32], and splenomegaly [8,9,11,13,16,20,21,33] were described in few of the manuscripts.

**Table 2:** Clinical features at birth (US, ultrasound; SNHL, sensorineural hearing loss).

References	Brain Abnormalities	Retinitis	SNHL	IUGR	Prematurity	Microcephaly	Thrombocytopenia	Hepatitis	Splenomegaly
**[7] (*n*./%)**	20/87	3/13	13/57	8/35	1/4	12/52	5/22	4/17	
**[8] (*n*./%)**	13/62				1/5		4/19		2/10
**[9] (*n*./%)**	139/88						9	4	19
**[10] (*n*./%)**	40/27								
**[11] (*n*./%)**	1/100		1/100	1/100	1/100	1/100	1/100	1/100	1/100
**[12] (*n*./%)**			1/100					1/100	
**[13] (*n*./%)**	9/69		11/85	4/31	2/15	5/38			2/15
**[14] (*n*./%)**	19/90	6/28	17/81	9/43		8/38	11/52	8/38	
**[15] (*n*./%)**	4/40		3/30					1/10	3/30
**[16] (*n*./%)**	1/100		1/100						
**[17] (*n*./%)**			1/100	1/100	1/100	1/100			
**[18] (*n*./%)**	1/100			1/100					
**[19] (*n*./%)**	1/100		1/100						
**[20] (*n*./%)**	71/83	3/3	29/34	39/45	46/53	31/36	72/84	46/53	45/52
**[21] (*n*./%)**	10/77	3/23	8/62	3/23		3/23	4/31	4/31	2/15
**[22] (*n*./%)**	10/63		16/100	4/25		2/13	3/19	4/25	
**[23] (*n*./%)**				1/100	1/100		1/100	1/100	
**[24] (*n*./%)**			7/100				3/43	2/29	
**[25] (*n*./%)**	10/83	4/33	9/75	4/33	8/67	4/33	5/42	5/42	
**[26] (*n*./%)**	23/88	7/27	21/81			8/31	12/46	10/38	
**[27] (*n*./%)**			59/100		4/7				
**[28] (*n*./%)**	1/100		1/100				1/100	1/100	
**[29] (*n*./%)**	0	0	0	0	0	0	0	0	0
**[30] (*n*./%)**	1/100	0	1/100						
**[31] (*n*./%)**	23/88	0	21/81	6/23		3/12	0	0	0
**[32] (*n*./%)**	11/31	0	6/17	6/17	2/6		2/6	1/3	0
**[33] (*n*./%)**	158/99	2/1	44/28	15/9		12/8	11/7	3/2	19/12

### 3.2. Treatment Plan and Side Effects (Table 3)

All studies included in our review used VGC in cCMV therapy (Table 3).

**Table 3:** Therapeutic protocol used (IV, intravenous; GC, Ganciclovir; bid, twice a day; w, weeks; VGC, Valganciclovir; od, once a day; SD, standard deviation; FOS, Foscarnet).

Ref.	Treatment Plan	Start of Therapy	Side Effects of VGC (*n*/%)
**[7]**	IV GC 5 mg/kg bid for 6 w, followed by oral VGC 17–18 mg/kg bid for 6 w, then od up to 1 year of age	First 2 weeks of life	Reversible neutropenia (12/52) Severe neutropenia (2/9) Central line infection (2/9)
**[8]**	IV GC 5 mg/kg bid for 6 w, followed by oral VGC 17 mg/kg bid for 6 w, then od up to 1 year of age OR oral VGC 17 mg/kg bid for 12 w, then od for 9 months	10.3 ± 7.8 months	Reversible neutropenia (11/52)
**[9]**	IV GC 5 mg/kg bid for 6 w, followed by oral VGC 17 mg/kg bid for 6 w, then od up to 1 year of age OR oral VGC 17 mg/kg bid for 12 w, then od up to 1 year of age		Reversible neutropenia (22/29)
**[10]**	IV GC 5 mg/kg bid for 6 w, followed by oral VGC 17 mg/kg bid for 6 w, then od up to 1 year of age OR oral VGC 17 mg/kg bid for 12 w, then od up to 1 year of age	First 4 weeks of life	Reversible neutropenia (33/22) Severe neutropenia (2/1)
**[11]**	IV GCV 6 mg/Kg bid for 6 w, followed by oral VGC 5 mg/Kg up to 15 mg/Kg bid for 10 w, then IV GC 6 mg/kg bid, and finally, FOS 180 mg/Kg/day for 6 w	Second day of life	Severe neutropenia (1/100) GCV/val-GCV resistance
**[12]**	IV GC 5 mg/kg bid for 1 w, followed by oral VGC 15 mg/kg bid for 5 w	17 days old	
**[13]**	IV GC 6 mg/kg bid for 3–6 w, followed by oral VGC 16 mg/kg bid for 3.5–12 months of age OR oral VGC 16 mg/kg bid for 3.5–12 months of age	Median 3 months (1.8–8.8 months)	Reversible neutropenia (6/46) Transiently raised aminotransferases (4/31)
**[14]**	Oral VGC 16 mg/kg bid for 6 w OR oral VGC 16 mg/kg bid for 6 months	4–77 days	Neutropenia (7/33)
**[15]**	IV GC OR oral VGC (dosage and duration not specified)	1–32 months	
**[16]**	IV GC 6 mg/kg bid for 2 w, followed by oral VGC 16 mg/kg bid for 4 w	5 days old	None
**[17]**	Oral VGC 17 mg/kg bid for 6 months for 13 w of age	4 months	Severe neutropenia (1/100)
**[18]**	Oral VGC 16 mg/kg bid for 6 w	5 months	Mild hepatitis (1/100) Reversible neutropenia (1/100)
**[19]**	Oral VGC 16 mg/kg bid for 12 w	5 w	None
**[20]**	Oral VGC 16 mg/kg bid for 6 w OR oral VGC 16 mg/kg bid for 6 months	First month of life	Reversable neutropenia (21/19) Severe neutropenia (3/3)
**[21]**	Oral VGC 15 mg/kg bid for 6 w	First month of life	Reversible neutropenia (1/8) Thrombocytopenia (1/8)
**[22]**	Oral VGC 16 mg/kg bid for 6 w OR oral VGC 16 mg/kg bid for 6 months	1–13.3 months	
**[23]**	IV GC 5 mg/kg bid for 4 w, followed by oral VGC 5 mg/kg bid for 6 w	Day 4 of life	
**[24]**	IV GC 5 mg/kg bid for 6 months OR oral VGC 16 mg/kg bid for 6 w	First 10 days of life	
**[25]**	Oral VGC (16–32 mg/kg/day) for 6–12 weeks and intravenous immunoglobulin (300 mg/kg/dose) twice within 2 weeks after the initiation of VGC	14 days	Reversible neutropenia (7/58) Genital bleeding (1/8)
**[26]**	Oral VGC (32 mg/kg/day) for 6 weeks (until June 2015) or 6 months	Median 12 days	Reversible neutropenia (9/24) Severe neutropenia (1/4) Thrombocytopenia (2/8) Genital bleeding (1/4) Impetigo (1/4) Hypocalcemia (1/4)
**[27]**	IV GV 5 mg/kg/d for 6 w, followed by oral VGC 17 mg/kg bid for another 6 w, then 1 daily dose until 12 months of treatment OR oral VGC 17 mg/kg bid for 12 weeks, then 1 daily dose until 12 months of treatment	First 12 weeks of life	Reversible neutropenia (29/49) Severe neutropenia (1/2)
**[28]**	IV GC 6 mg/kg bid for 6 w, followed by oral VGC 56 mg/kg per day for another 6 w	Day 4 of life	
**[29]**	Oral VGC 15 mg/kg bid for 6 w	6 months	None
**[30]**	IV GCV 6 mg/kg bid for 6 w, followed by oral VGC 11 mg/kg bid for another 6 w	1.5 months of age	None
**[31]**	Oral VGC 16 mg/kg bid for 6 months	9.5 months (range 0–46)	Reversible neutropenia (6/23)
**[32]**	Oral VGC 16 mg/kg bid for 6 w (or other 6-week therapy cycles if viremia was found positive) OR oral VGC 16 mg/kg bid for 6 months (after 2015)	4.23 years ± 1.57 SD	Reversible neutropenia (1/3)
**[33]**	Oral VGC 17 mg/kg bid for 12 w, then 1 daily dose until the age of 1 year	First 4 weeks after birth	Reversible neutropenia (46/29) Severe neutropenia (7/4) Reversible anemia (12/8)

The protocol used was very different between the different studies, especially before publication of the results reported by Kimberlin in 2015 [20].

Fourteen studies used intravenous GCV before oral VGC [7,8,9,10,11,12,13,15,16,23,24,27,28,30]. In all of these manuscripts, the doses and durations of therapy varied widely. Most studies administered GC at a dose of 5 mg/kg [7,8,9,10,27] or 6 mg/kg [11,28,30] twice per day for 6 weeks. The study by Çiftdoğan et al. [12] used it only for one week, that by Del Rosal et al. [13] used it for periods of time ranging from 3 to 6 weeks with a dose of 6 mg/kg twice per day, that by Hayakawa et al. [16] used a 6 mg/kg dose twice per day for 2 weeks, that by Muller et al. [23] used a 5 mg/kg dose twice per day for 4 weeks, and that by Mazzaferri et al. [24] administered it for 6 months.

The IV GC treatments were followed by the oral administration of VGC, but again, the doses and durations of therapy were highly variable. Most used VGC at a dose of 17 mg/kg bid for 6 weeks, followed by once daily for up to one year [7,8,9,10,27]. The study by Çiftdoğan et al. [12] used VGC at a dose of 15 mg/kg twice per day for 5 weeks, that by Del Rosal et al. [13] used a 16 mg/kg dose twice per day for 3–12 months, that by Hayakawa et al. [16] used a 16 mg/kg dose twice per day for 4 weeks, that by Muller et al. [23] used a 5 mg/kg dose twice per day for 6 weeks, that by Pasternak et al. [28] used a 56 mg/kg dose daily for another 6 weeks, and that by Suganuma et al. [30] used an 11 mg/kg dose twice per day for another 6 weeks.

Especially after the study by Kimberlin et al. [20] was published, twenty manuscripts [8,9,10,13,14,15,17,18,19,20,21,22,24,25,26,27,29,31,32,33] used VGC without the intravenous administration of GC. Again, the durations and the doses differed between the studies.

Most studies have administered VGC at a dose of 16–17 mg/kg bid for 6 months [14,17,20,22,26,31,32] or for 6 weeks [14,18,20,21,22,24,26,29,32]. Others have used it at a dose of 16–17 mg/kg bid for 12 weeks, then once per day until one year of age [8,9,10,13,27,33].

The study by Kashiwagi et al. [19] administered VGC for 12 weeks and that by Nishida et al. [25] associated intravenous immunoglobulins in the first 2 weeks.

The study by Campanini et al. [11] used a particular protocol due to the emergence of resistance to antiviral therapy, while the study by Gabbay-Ben Ziv et al. [15] did not specify the doses and the durations of the therapies used.

Finally, the therapeutic protocols of the various studies were also distinguished by the starting dates of the drugs. In fact, they ranged from the first weeks of life [7,10,11,12,14,16,19,20,21,23,24,25,26,28] to several months after birth [8,13,15,17,18,22,27,29,30,31,32,33].

In terms of side effects, the most frequent was mild reversible neutropenia, found in a total of 15 studies. The percentage was highly variable, ranging from 8 to 100% of the patients included. In some cases, the neutropenia was so severe as to require, in addition to the suspension of the VGC, the administration of growth factors [7,10,11,17,20,26,27,33].

Other reported rare side effects were hepatitis [13,18], thrombocytopenia [21,26], genital bleeding [25,26], and anemia [33].

### 3.3. Outcome after Treatment (Table 4)

Out of 27 studies, 24 analyzed the outcomes after the treatment with VGC.

Ten studies [7,10,12,13,15,18,20,21,24,25] showed reductions in the numbers of children with SNHL after therapy was performed. In five of the manuscripts [16,17,19,28,30], this number remained unchanged, even when they were all case reports.

Considering the patients whose ears had defects before the start of therapy, 13 studies showed reductions in the numbers of organs with defects after VGC [7,8,10,13,15,17,18,20,24,25,27,29,31], while 3 case reports [16,19,30] showed no improvements. In particular, four studies [10,25,26,32] reported deteriorations in normal patient ears after therapy, which were rare events because most of the organs did not show changes, and the percentages of affected patients ranged from 87 to 100%. In fact, most of the abnormal patient ears improved or showed no changes with therapy. Five studies [7,10,20,25,26,27,32] reported further deterioration, with the percentages ranging from 5 to 11%. 

Only the study by McCrary et al. [22] reported different results, with a total of 20 (63%) abnormal patient ears further worsening after treatment with VGC.

Thirteen studies analyzed neurodevelopmental outcomes in children with cCMV who underwent treatment. No impairments were reported in five manuscripts [15,16,17,19,30].

The study by Fukushima et al. [14] showed no impairments in 29% of children, mild sequelae in 19%, and severe sequelae in 52%.

In their case reports, Imamura et al. [18] reported severe neurodevelopmental delays, while Müller et al. [23] reported mild neurodevelopmental delays.

The study by Kimberlin et al. [20] demonstrated that a group treated for an extended period of 6 months, compared with a 6-week group, had higher neurodevelopmental rating scale scores at 24 months.

Nishida et al. [25] showed severe impairments in 33% of patients, mild impairments in 25%, and normal development in 42% of children.

The study by Turriziani Colonna et al. [32] reported normal development in 91% of cases, speech disorders in 19%, and pathological internalization scales in 25% of children.

**Table 4:** Outcomes after treatment (SNHL, sensorineural hearing loss; mo, months; wk, weeks; GC, Ganciclovir; VGC, Valganciclovir).

Ref.	Babies with SNHL before Treatment	Babies with SNHL after Treatment	Ears with Defects before Treatment	Ears with Defects after Treatment	Normal Ears after Treatment	Abnormal Ears after Treatment	Neurodevelopmental Outcomes after Treatment
**[7] (*n*./%)**	13/57	8/39	21/46	11/24	No change: 25/100 Worsening: 0	Improved: 12/57 No change: 8/38 Worsening: 1/5	
**[8] (*n*./%)**	21/100		Mild: 22/52 Moderate: 10/24 Severe: 3/7	Mild: 2/5 Moderate: 2/5 Severe: 2/5	No change: 7/100 Worsening: 0	Improved: 29/83 No change: 13/31 Worsening: 0	
**[9] (*n*./%)**	110						
**[10] (*n*./%)**	24/16	12/8	Mild: 36/12 Moderate: 19/6 Severe: 22/7	Mild: 14/5 Moderate: 9/3 Severe: 17/6	No change: 124/99 Worsening: 1/1	Improved: 50/65 No change: 22/29 Worsening: 5/7	
**[11] (*n*./%)**	1/100						
**[12] (*n*./%)**	1/100	0					
**[13] (*n*./%)**	11/85	6/46	Mild: 7/27 Moderate: 3/12 Severe: 8/31	Mild: 3/12 Moderate: 1/4 Severe: 7/27	No change: 8/100 Worsening: 0	Improved: 7/39 No change: 11/61 Worsening: 0	
**[14] (*n*./%)**							No impairment: 6/29 Mild sequelae: 4/19 Severe sequelae: 11/52
**[15] (*n*./%)**	3/30	1/10	4/20	1/5	No change: 4/100 Worsening: 0	Improved: 3/75 No change: 1/25 Worsening: 0	No impairment: 4/100
**[16] (*n*./%)**	1/100	1/100	2/100	2/100		Improved: 1/50 No change: 1/50 Worsening: 0	No impairment: 1/100
**[17] (*n*./%)**	1/100	1/100	2/100	1/50		Improved: 1/50 No change: 1/50 Worsening: 0	No impairment: 1/100
**[18] (*n*./%)**	1/100	0	1/50	0	No change: 1/100 Worsening: 0	Improved: 1/100 No change: 0 Worsening: 0	Severe neurodevelopmental delay: 1/100
**[19] (*n*./%)**	1/100	1/100	2/100	2/100		Improved: 1/50 No change: 1/50 Worsening: 0	No impairment: 1/100
**[20] (*n*./%)**	6 mo group: 11/26 6 wk group: 18/42	6 mo group: 7/19 6 wk group: 11/35	6 mo group: 36/42 6 wk group: 45/52	6 mo group: 22/31 6 wk group: 23/40	6 mo group: 48/69 6 wk group: 35/60	Improved: 6 mo group: 6/9 6 wk group: 2/3 No change: 6 mo group: 8/11 6 wk group: 16/28 Worsening: 6 mo group: 8/11 6 wk group: 5/9	6 mo group, compared with 6 wk group, had higher Bayley-III language-composite scores and receptive-communication scale scores at 24 months
**[21] (*n*./%)**	Mild: 0 Moderate: 5/38 Severe: 3/23	Improved: 2/25 No change: 6/75 Worsening: 0			No change: 5/100 Worsening: 0		
**[22] (*n*./%)**	16/100		32/100			Improved: 6/19 No change: 6/19 Worsening: 20/63	
**[23] (*n*./%)**		0					Mild neurodevelopmental delay: 1/100
**[24] (*n*./%)**	7/100	GC group: 3/75 VGC group: 2/67	13/93	GC group: 5/63 VGC group: 1/17	No change: 1/100 Worsening: 0	Improved GC group: 3/38 VGC group: 6/100 No change GC group: 4/50 VGC group: 0 Worsening GC group: 0 VGC group: 0	
**[25] (*n*./%)**	9/75	6/50	16/67	9/38	No change: 7/88 Worsening: 1/13	Improved: 8/50 No change: 7/44 Worsening: 1/6	Severe impairment: 4/33 Mild impairment: 3/25 Normal development: 5/42
**[26] (*n*./%)**	21/81		Severe: 16/31 Moderate: 13/25 Mild: 8/15		No change: 20/87 Worsening: 3/13	Improved: 16/55 No change: 11/38 Worsening: 2/7	
**[27] (*n*./%)**	59/100		Mild: 41/35 Moderate:20/17 Severe: 19/16	Mild: 3/7 Moderate: 6/5 Severe: 18/15	No change: 38/100 Worsening: 0	Improved: 55/47 No change: 26/22 Worsening: 2/2	
**[28] (*n*./%)**	1/100	1/100					
**[29] (*n*./%)**	1/100		2/100	0		0	
**[30] (*n*./%)**	1/100	1/100	1/50	1/50	No change: 1/100 Worsening: 0	Improved: 0 No change: 1/100 Worsening: 0	Normal development: 1/100
**[31] (*n*./%)**	21/81		38/73	36/69	No change: 5/100 Worsening: 0	Improved: 9/24 No change: 29/76 Worsening: 0	
**[32] (*n*./%)**	6/17		10/14		No change: 56/96 Worsening: 2/4	Worsening: 2	Normal development: 30/91 Borderline score: 3/9 Language disorders: 6/19 Pathological internalizing scale: 7/25 Autism spectrum disorder: 0
**[33] (*n*./%)**	Not investigated						

## 4. Discussion

cCMV is an infectious disease that continues to present a series of gray areas regarding diagnosis, prognosis, and therapy.

The condition is important, and although it is a rare disease, it has a higher frequency than the most common congenital disease diagnosed with the newborn screening, i.e., congenital hypothyroidism [34]. The birth prevalence of congenital CMV infection is 0.64%, according to Kenneson and Cannon [1].

Furthermore, it is an important cause of deafness. In the United States, cCMV causes 21% of deafness identified at birth and 25% of that present at 4 years of age [35].

Most children with congenital CMV infection—approximately 85–90% [36]—have no clinical findings at birth (asymptomatic infection). In the remaining 10–15%, at birth, it is possible to identify the involvement of the central nervous system, with microcephaly, radiographic anomalies indicative of CMV disease, chorioretinitis, hearing impairment, or the involvement of other organs, along with thrombocytopenia, petechiae, hepatomegaly, splenomegaly, IUGR, and hepatitis [37]. Approximately 10% of asymptomatic children will develop SNHL; however, the clinical, laboratory, or instrumental characteristics capable of predicting which asymptomatic child will develop hearing loss are still unknown [37].

The children with cCMV included in our review presented the clinical features already described in the literature, confirming that in symptomatic patients, the most frequent anomalies were brain abnormalities and SNHL, while the rare anomalies were ocular ones.

The possibility that an asymptomatic newborn may develop SNHL in the future without having any premonitory signs is a challenge. According to the 2007 position statement of the Joint Committee on Infant Hearing, all hearing-impaired infants are advised to undergo hearing screening at 1 month of age to detect hearing loss at 3 months and to start rehabilitation at 6 months [38].

Regarding drug treatment, in 2003, Kimberlin et al. demonstrated that GV therapy in symptomatic infants with cCMV involving the CNS prevented hearing worsening at 6 months and 1 year of age [39]. They subsequently reported that an oral VGC 16 mg/kg dose provided the same systemic exposure as an IV Ganciclovir 6 mg/kg dose [40]. Finally, in 2015, they demonstrated that prolonged VGV therapy for 6 months did not ameliorate short-term hearing loss, but it improved modest hearing loss and development in the long-term [20].

Table 4 describes the outcomes reported after treatment. Neonatal lesions are likely irreversible, which explains why antiviral therapy is only moderately effective and serves to prevent further deterioration. In fact, in cases of mild SNHL, in general, the manuscripts reported improvements, while for severe forms, no changes or worsening were observed. Due to the rarity of cCMV and the even more rare severe SNHL, conclusive evidence cannot be obtained.

The therapy proved to be well-tolerated, with most of the side effects attributable to the intravenous administration of GCV. Among the non-negligible side effects of oral VGC, we observed the possible development of neutropenia, which was reversible with dose reductions or drug suspensions, and which only occurred in isolated cases as it required the use of growth factors (Table 3).

Consequently, following publication of the Kimberlin studies, the 2017 consensus recommended treating infants with symptomatic congenital CMV disease with oral VGC for 6 months (16 mg/kg dose twice per day) within the first month of life. They reiterated not treating children with asymptomatic congenital CMV infection, not routinely treating children with “mild symptomatic” congenital CMV disease (e.g., transient thrombocytopenia or isolated IUGR), and not routinely treating children with isolated sensorineural hearing loss [41].

Due to improved awareness, increased maternal screenings and (targeted) newborn screenings, and the better availability of VGCs, in recent years, there has been an increase in the diagnosed and treated cases of cCMV with few or no symptoms (e.g., isolated SNHL). As can be seen from the studies reported in our review, these children are also treated with VGC, many of them for 6 months or more and with different protocols, e.g., GCV in addition to VGC, different doses, different durations of therapies, and variable starts of the drug. This creates a series of problems and difficulties because there is still no definitive evidence for the best treatment for these children; randomized placebo-controlled trials continue to be very difficult due to a common belief in the efficacy of the therapy; there is a lack of virological support for long-term treatment, a lack of biomarkers of viral clearance, and in some cases, there are no persistent localized immunological responses; our knowledge of the pathogenesis and reversibility of long-term impairment is incomplete; and drugs (with potential side effects) should not be administered without first being validated by scientific studies, and the creation of potential false hopes in parents, the induction of antiviral resistance, and the costs of long-term treatment should be minimized.

Our review has several limitations. The quality of the included studies and the sample size were limited due to the relative rarity of the disease. The use of different therapeutic protocols in terms of starting dates, doses, and durations made it impossible to compare and correctly evaluate the efficacy of the treatment. An important limitation was that most of the manuscripts included (14/27) presented a follow-up of 6–12 months. As evidenced by the study by Fowler et al. [2], cCMV is able to cause delayed SNHL in approximately 20% of children, with the age of onset ranging from 25 to 62 months. This may justify McCrary’s findings compared to the other studies. Consequently, although difficult, it is imperative that future studies on the effect of VGC will include a follow-up at 5–6 years of age to evaluate the effect of therapy on fluctuating SNHL. Nearly all studies reported only ABR results without tympanometry, not excluding the presence or lack of an effusion, which is a frequent occurrence at this age. Finally, the use of only open access articles and the initial selection by abstracts may have led to the exclusion of some articles.

## 5. Conclusions

In conclusion, our review showed that the use of VGC in children with cCMV of differing levels of severity is growing, with the drug being mostly well-tolerated or with side effects that are reversible with drug suspension. However, the protocols used were diverse, and this limited their interpretation. However, a trend toward potential good effects on SNHL in some of the cohorts suggests that new studies with better-characterized cohorts will still be worthwhile. For example, early and sustained viral suppression may be related to better hearing outcomes. Consequently, in our opinion, a future antiviral treatment should monitor both the CMV viral load in the blood and the CMV-related immune system responses, both systemically and localized (e.g., in the inner ear), stopping the treatment only when the adaptive immune response is able to control the viral load. In addition, the follow-up period must be prolonged due to the characteristics of fluctuating SNHL.

## Figures and Tables

**Table 1:** Studies included in the review.

Reference	Country	Study Design	*N*. of Patients	Months of Follow-Up
**Amir**, **2010 [7]**	Israel	Retrospective study	23	12
**Amir**, **2013 [8]**	Israel	Retrospective study	21	12
**Bilavsky**, **2015 [9]**	Israel	Retrospective study	210	12
**Bilavsky**, **2016 [10]**	Israel	Retrospective study	149	12
**Campanini**, **2012 [11]**	Italy	Case report	1	6
**Çiftdoğan**, **2011 [12]**	Turkey	Case report	1	12
**Del Rosal**, **2012 [13]**	Spain	Retrospective case series	13	12
**Fukushima**, **2019 [14]**	Japan	Prospective cohort study	21	18
**Gabbay-Ben Ziv**, **2012 [15]**	Israel	Retrospective cohort study	10	1–62
**Hayakawa**, **2012 [16]**	Japan	Case report	1	12
**Hilgendorff**, **2009 [17]**	Germany	Case report	1	60
**Imamura**, **2011 [18]**	Japan	Case report	1	12
**Kashiwagi**, **2011 [19]**	Japan	Case report	1	6
**Kimberlin**, **2015 [20]**	United States	Randomized controlled trial	86	24
**Lombardi**, **2009 [21]**	Italy	Prospective cohort study	13	6
**McCrary**, **2019 [22]**	United States	Retrospective study	16	38
**Muller**, **2008 [23]**	Germany	Case report	1	6
**Mazzaferri**, **2017 [24]**	Italy	Retrospective study	7	24
**Nishida**, **2016 [25]**	Japan	Prospective study	12	36
**Ohyama**, **2019 [26]**	Japan	Prospective study	26	6
**Pasternak**, **2018 [27]**	Israel	Retrospective study	59	≥12
**Schulzke**, **2006 [28]**	Switzerland	Case report	1	9
**Stronati**, **2011 [29]**	Italy	Case report	1	60
**Suganuma**, **2018 [30]**	Japan	Case report	1	120
**Suganuma**, **2020 [31]**	Japan	Retrospective study	26	Not specified
**Turriziani Colonna**, **2020 [32]**	Italy	Retrospective study	36	48
**Ziv**, **2018 [33]**	Israel	Retrospective study	160	60

## Data Availability

The data are available upon reasonable request to the corresponding author.
